# Substrate uptake patterns shape niche separation in marine prokaryotic microbiome

**DOI:** 10.1126/sciadv.adn5143

**Published:** 2024-05-15

**Authors:** Zihao Zhao, Chie Amano, Thomas Reinthaler, Mónica V. Orellana, Gerhard J. Herndl

**Affiliations:** ^1^ Department of Functional and Evolutionary Ecology, Bio-Oceanography and Marine Biology Unit, University of Vienna, Djerassiplatz 1, A-1030 Vienna, Austria.; ^2^ Polar Science Center, Applied Physics Laboratory, University of Washington, Seattle, WA 98195, USA.; ^3^ Institute for Systems Biology, Seattle, WA 98109, USA.; ^4^ NIOZ, Department of Marine Microbiology and Biogeochemistry, Royal Netherlands Institute for Sea Research, Den Burg, Netherlands.; ^5^ Environmental and Climate Research Hub, University of Vienna, Althanstraße 14, A-1090 Vienna, Austria.

## Abstract

Marine heterotrophic prokaryotes primarily take up ambient substrates using transporters. The patterns of transporters targeting particular substrates shape the ecological role of heterotrophic prokaryotes in marine organic matter cycles. Here, we report a size-fractionated pattern in the expression of prokaryotic transporters throughout the oceanic water column due to taxonomic variations, revealed by a multi-“omics” approach targeting ATP-binding cassette (ABC) transporters and TonB-dependent transporters (TBDTs). Substrate specificity analyses showed that marine SAR11, Rhodobacterales, and Oceanospirillales use ABC transporters to take up organic nitrogenous compounds in the free-living fraction, while Alteromonadales, Bacteroidetes, and Sphingomonadales use TBDTs for carbon-rich organic matter and metal chelates on particles. The expression of transporter proteins also supports distinct lifestyles of deep-sea prokaryotes. Our results suggest that transporter divergency in organic matter assimilation reflects a pronounced niche separation in the prokaryote-mediated organic matter cycles.

## Teaser

Particle-associated and free-living prokaryotes exhibit distinct substrate preferences throughout the oceanic water column.

## INTRODUCTION

Marine organic matter is synthesized in the surface ocean primarily by phytoplankton and transported into the ocean interior as particulate organic matter (POM) or dissolved organic matter (DOM) organic matter through convection, diffusion, and water mass mixing (*1*, *2*). The incorporation, redistribution, and remineralization of organic matter, however, are mediated mainly by hetero(organo)trophic prokaryotes (bacteria and archaea) (*3*, *4*). Most heterotrophic prokaryotes can only take up molecules smaller than 600 Da (*5*). Therefore, POM and high–molecular weight DOM need to be hydrolyzed before assimilation (*5*). In addition, the substrate specificity of membrane-bound transporter protein systems determines substrate uptake even if the size of the molecule is theoretically small enough to be transferred into the cell (*6*–*13*).

ATP-binding cassette (ABC) transporters and TonB-dependent transporters (TBDTs) are the two major types of transporters for active exchange of substrate between the ambient marine environment and the prokaryotic cell (*13*–*15*). ABC transporters can import organic nitrogen such as ammonium/urea and amino acids/peptides, sugars, phosphorus/phosphonate, as well as metal-chelate complexes (*15*). TBDTs transport mono- and polysaccharides and play an important role in iron/siderophore and vitamin B_12_ uptake (*13*, *14*). Because DOM is generally refractory in the deep ocean (*16*, *17*), deep-sea heterotrophic prokaryotes likely primarily solubilize POM to cover their carbon and energy requirements (*18*, *19*). Thus, the secretion of extracellular enzymes (*20*, *21*), the following uptake of substrate by transporters (*8*, *9*), together with cell production and activity (*18*, *22*) support the hypothesis that deep-sea prokaryotic activity is primarily particle associated.

However, the question still remains how prokaryotes use transporters to assimilate substrate in the water column. The shifts between free-living (FL) and particle-associated (PA) lifestyles might also change the expression level of transporter proteins because genetic evidence suggests that deep-sea PA prokaryotes have distinct substrate assimilation/remineralization features (*23*, *24*). Yet, direct comparison on the substrate variability of prokaryotic transporters in different size fractions is limited. Particularly, in contrast to ABC transporters, the substrate specificity of TBDTs is poorly documented for the marine environment especially in the deep ocean (*8*, *11*, *13*, *14*, *25*). This knowledge gap precludes a mechanistic understanding of the role of prokaryotes in marine organic matter cycles.

Here, we combined metagenomic, metatranscriptomic and metaproteomic analyses and focused on the major variations (depth stratification and size fractionation) in ABC transporters and TBDTs of marine prokaryotic communities. We hypothesized that the genetic potential, transcriptional response, and proteomic expression of transporter proteins reflect how prokaryotes adapt to FL or PA lifestyles in different depth layers. Changes in substrate specificity of both ABC transporters and TBDTs suggest variability in substrate bioavailability and provide further insights into the niche separation of prokaryotes in the marine organic matter cycling.

## RESULTS AND DISCUSSION

### Distinct profiles of marine prokaryotic transporters revealed by metagenomics, metatranscriptomics and metaproteomics

**Fig. 1.**
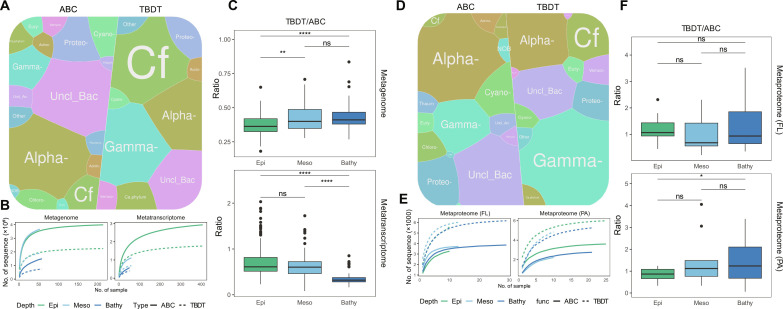
Profile of microbial ABC transporters and TBDTs in metagenomic, metatranscriptomic, and metaproteomic dataset. (**A**) Occurrence of genes encoding ABC transporters and TBDTs in different taxonomic groups. (**B**) Rarefication curves for genes/transcripts encoding ABC transporters and TBDTs throughout water column. (**C**) Ratio of TBDT/ABC in metagenomic and metatranscriptomic dataset. (**D**) Occurrence of proteins encoding ABC transporters and TBDTs in different taxonomic groups. (**E**) Rarefication curves for ABC transporters and TBDTs throughout water column. (**F**) Ratio of TBDT/ABC in FL and PA metaproteomes. Box shows median and interquartile range (IQR); whiskers show 1.5 × IQR of the lower and upper quartiles or range; outliers extend to the data range. Statistics are based on Wilcoxon test. *P < 0.05, **P < 0.01, and ****P < 0.0001. ns, not significant. Alpha-, Alphaproteobacteria; Gamma-, Gammaproteobacteria; Cf, Bacteroidetes; Thaum, Thaumarchaea; Eury-, Euryarchaea; NOB, Nitrospinae; Cloro-, Cloroflexi; Firmi-, Firmicutes; Ca.phylum, candidatus phylum; Proteo-, unclassified Proteobacteria; Uncl_Bac, unclassified bacteria; Uncl_Arc, unclassified archaea.

By examining 105,585,296 sequences from metagenomic assemblies (*26*), we identified 479,317 and 272,981 sequences encoding ABC transporters and TBDTs of prokaryotes (Fig. 1A and table S1), respectively. Taxonomic assignment to transporter genes indicated that genes encoding ABC transporters were dominated by Alphaproteobacteria (29%) (Fig. 1A). Unclassified bacteria (27%) and unclassified Proteobacteria (9%), Gammaproteobacteria (8%), and Bacteroidetes (5%) were also major taxonomic groups contributing to the gene pool of ABC transporters. However, of the genes encoding TBDTs, about 50% originated from Bacteroidetes (27%) and Gammaproteobacteria (23%), followed by Alphaproteobacteria (17%) (Fig. 1A). Rarefaction analysis showed that the richness of genes and transcripts encoding ABC transporters is higher than that of TBDTs (Fig. 1B), but the sequence richness decreased with depth for both types of transporters in the metagenomic and metatranscriptomic dataset (Fig. 1B). To evaluate the relative abundance of genes and transcripts encoding ABC transporters and TBDTs, reads from metagenomic and metatranscriptomic samples collected from the entire water column (dataset S1) were mapped to the corresponding genes. A significantly higher relative abundance of transporters (both ABC transporters and TBDTs) was found in the bathypelagic than in the epipelagic realm in both the metagenome and metatranscriptome (fig. S1, A and B). Examining the ratio of TBDTs and ABC transporters (TBDT/ABC), major differences were found between the metagenomic and metatranscriptomic dataset (Fig. 1C). In the metagenome, the prokaryotic community in the deep ocean exhibited a higher TBDT/ABC ratio than the epi- and mesopelagic prokaryotic community, indicating a genetic preference of prokayotes in using TBDTs for substrate uptake. In the metatranscriptome, however, the TBDT/ABC ratio decreased toward deeper waters, which suggests that the deep-sea prokaryotes express more transcripts encoding ABC transporters than TBDTs. This indicates that the bioavailable substrates might be mainly assimilated by ABC transporters in the deep ocean. The discrepancy between genetic potential and transcriptional response might be caused by either depth-related community stratification of both total and active prokaryotes (*24*, *27*–*29*) or the variability of substrate concentration and availability throughout the water column (*17*, *18*), where the metabolic response of the active community shifts accordingly (*30*).

Why do deep-sea prokaryotes carry more TBDT genes but tend to use ABC transporters for substrate uptake? The TBDT/ABC transporter ratio in the metatranscriptome might be affected by sample handling time because the half-life time of mRNA is rather short (*31*, *32*), and the depressurization process may change prokaryotic metabolism (*22*). Proteins are more stable compared to mRNA. Thus, we examined 48 metaproteomic samples from 22 open ocean stations with at least two technical replicates (dataset S1) and focused on the expression of ABC transporters and TBDTs. It has been suggested that deep-sea prokaryotes are PA due to the refractory nature of DOM (*16*–*18*, *20*). Hence, differences in lifestyle may also affect the patterns of substrate assimilation. We distinguished FL prokaryotes in the size range of 0.2 to 0.8 μm from PA prokaryotes retained on 0.8-μm pore size filters.

On the basis of protein analyses, we identified 4531 ABC transporters and 7225 TBDTs (table S1). The ABC transporters and TBDTs in the metaproteomes and metagenomes were of similar taxonomic origin. ABC transporters and TBDTs mainly originated from Alphaproteobacteria, Gammaproteobacteria, and Bacteroidetes (Fig. 1D). Consistent with gene analysis, alphaproteobacterial ABC transporter showed highest protein occurrence (33%), followed by Gammaproteobacteria (13%). TBDTs were mainly of gammaproteobacterial origin (42%), and Bacteroidetes TBDTs showed lower occurrence (10%) in the metaproteome than the corresponding genes in the metagenome (27%), which makes Alphaproteobacteria (19%) the second most abundant group for TBDT proteins. The richness of ABC transporters and TBDTs, however, was very different in the metaproteomes compared to the corresponding genes/transcripts from the metagenomes and metatranscriptomes (Fig. 1E). In the metaproteomes, TBDTs exhibited a higher richness than ABC transporters in both the FL and PA prokaryotes. For the FL prokaryotes, the relative protein abundance of ABC transporters and TBDTs was similar throughout the water column (fig. S1C), resulting in a relatively constant ratio of TBDT/ABC (Fig. 1F). For the PA prokaryotes, the relative protein abundance of ABC transporters was lower in the meso- and bathypelagic waters, while the relative protein abundance of TBDTs was higher (fig. S1D). The ratio of TBDT/ABC transporters in the PA fraction in the bathypelagic, however, was only significantly higher than in the epipelagic waters (Fig. 1F; Wilcoxon test, *P* < 0.05). This indicates that TBDTs might facilitate substrate uptake in the PA prokaryotic community in the deep ocean. Deep-sea prokaryotes maintain a high capacity to secrete extracellular enzymes for hydrolyzing POM for carbon and energy acquisition (*20*). The hydrolysis products from enzymatic cleavage of POM can be directly assimilated by PA prokaryotes through TBDTs. This suggests that, although deep-sea prokaryotes preferentially carry genes encoding TBDTs (Fig. 1C), the expression of those genes is rather depending on the environmental condition (Fig. 1F) and that changes in lifestyles apparently strongly influence the physiological response of deep-sea prokaryotes.

### Depth-related community variation marginally affects prokaryotic utilization of transporters

**Fig. 2.**
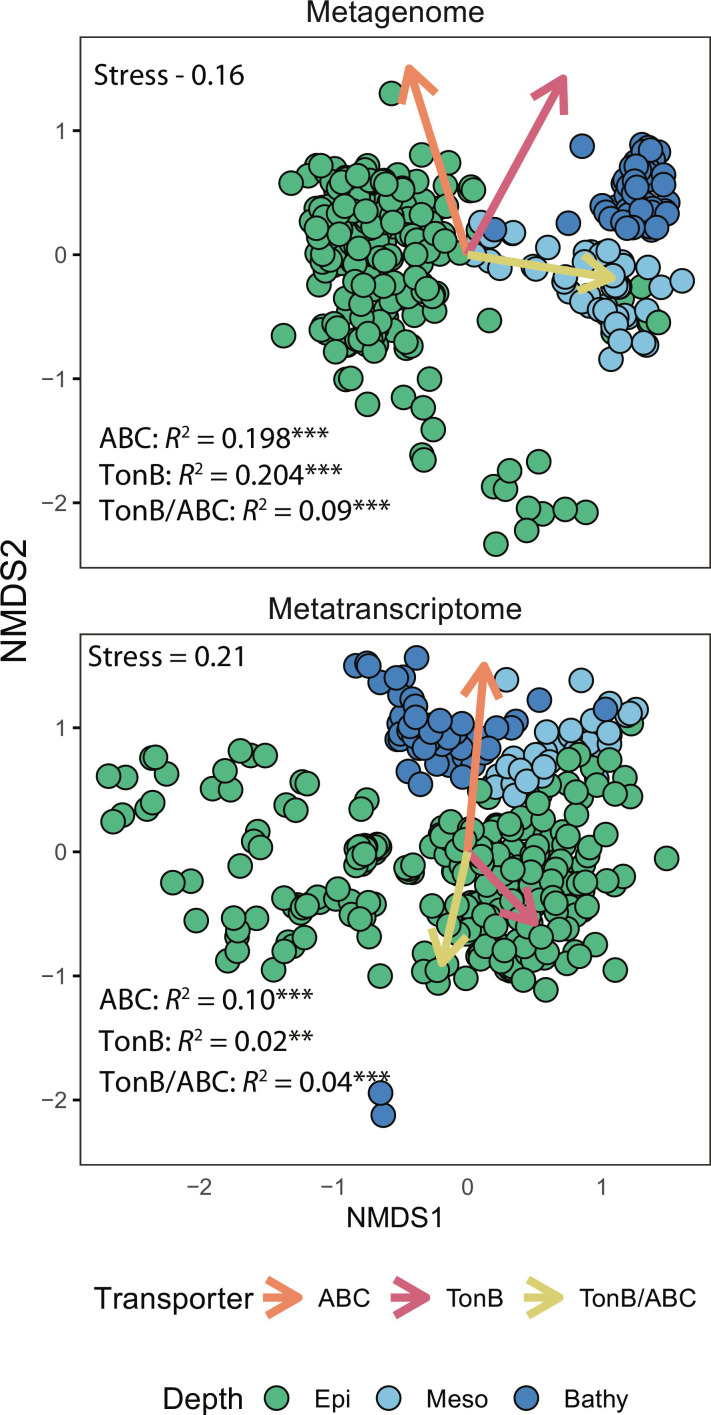
NMDS plot revealing the weak correlation between the gene/transcript abundances of ABC transporters and TBDTs with the mOTU-based community structure. The abundance of ABC transporters and TBDTs and the ratio of TBDTs/ABC transporters (arrows) were fitted to community structures (points). **P < 0.01 and ***P < 0.001.

We examined the relationship between depth-related community variation, as reflected by phylogenetic marker gene-based operational taxonomic units (mOTUs) (*33*), and the changes of genes/transcripts encoding ABC transporters and TBDTs. In NMDS plots, the community structure of the total and the active community (i.e., the prokaryotic communities deduced from metagenome and metatranscriptome data, respectively) was only weakly related to the abundance of ABC transporters and TBDTs [permutational analysis of variance, coefficient of determination (*R*^2^) < 0.2, *P* < 0.05; Fig. 2]. Similarly, no significant correlations between the prokaryotic diversity (Shannon index) and the transporter profiles (gene/transcript abundance and their corresponding TBDT/ABC ratio) were found (fig. S2A). The covariance between community structure based on Bray-Curtis dissimilarity matrices and transporter composition (abundance and ratio) from Euclidean distances was also weak in all depth layers (fig. S2B). This suggests that the utilization/expression of transporters is only weakly related to depth stratification in prokaryotic community composition (*27*). Instead, substrate availability might be one of the responsible factors for variations in the transporter utilization and expression at the genetic and metabolic level because organic matter availability is decreasing with depth in the water column (*34*).

### Taxonomic origin and substrate specificity of prokaryotic ABC transporters are size-fractionated throughout the water column

**Fig. 3.**
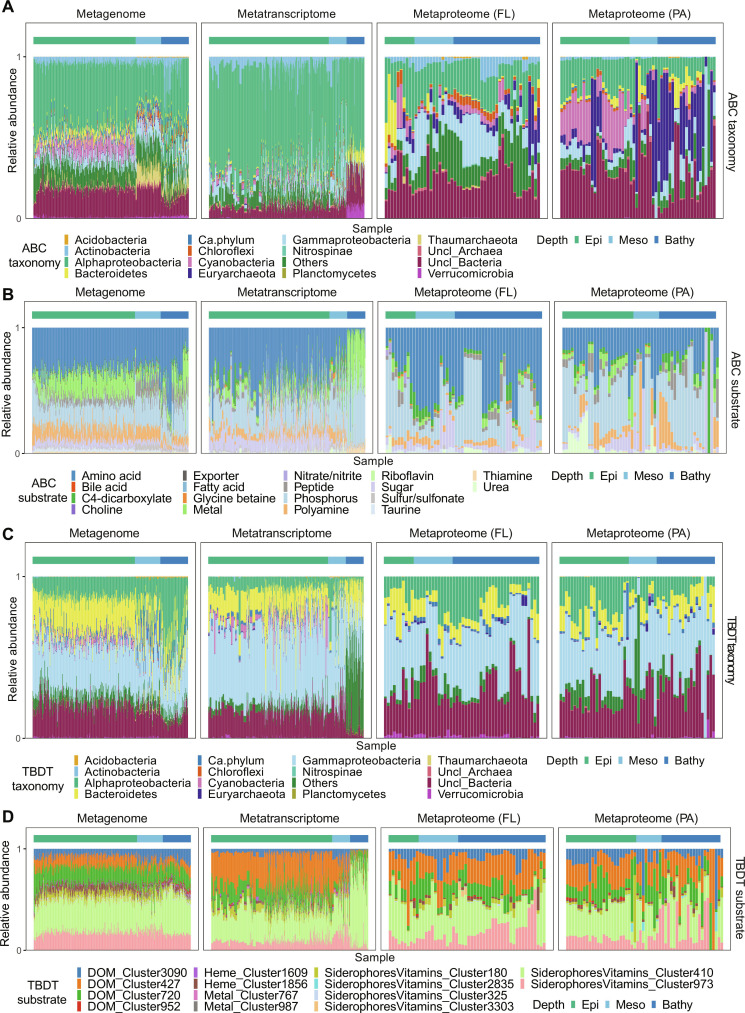
Taxonomic breakdown and substrate specificity of ABC transporters and TBDTs in metagenomic, metatranscriptomic, and metaproteomic dataset. (**A**) Taxonomic classification of ABC transporters. (**B**) Substrate specificity of ABC transporters. (**C**) Taxonomic classification of TBDTs. (**D**) Substrate specificity of TBDTs. The *x* axis represents samples from different stations, and the depth layers of the sample are indicated using the color bar on the top. The horizontal color bar refers to sampling depth as defined in Fig. 1C.

By examining the taxonomic affiliation of genes, transcripts, and proteins of ABC transporters, we found that Alphaproteobacteria dominated the ABC transporter gene/transcript pool throughout the water column (21 to 40% in the metagenome and 57 to 60% in the metatranscriptome; Fig. 3A and dataset S2). Cyanobacteria also carried genes encoding ABC transporters (~7%) in the epipelagic, but their transcripts were relatively low (<2%; Fig. 3A and dataset S2). In the metaproteome, although alphaproteobacterial and cyanobacterial ABC transporters were still dominating, clear differences were found between size fractions for ABC transporters in these two groups. In the FL fraction, ABC transporters were dominated by Alphaproteobacteria and Gammaproteobacteria. Cyanobacterial ABC transporters were mainly found in the PA fraction (22 ± 10%, >0.8 μm; dataset S2) in the epipelagic due to their large cell size (*35*).

A profound depth-related taxonomic variation was found in ABC transporters in the PA fraction. A fairly high contribution of Euryarchaea to ABC transporters was detected in the metaproteome especially in the PA fraction in the deep ocean (35 ± 23%; Fig. 3A and dataset S2). We further compared the relative abundance of Euryarchaea in the metagenome, metatranscriptome, and metaproteome (fig. S3A). We found that the relative abundance of euryarchaeal genes and transcripts (based on mOTUs) was two orders of magnitude lower than the relative abundance of euryarchaeal proteins (fig. S3A). This suggests that Euryarchaea might have a high protein content, which might allow the expression of large amounts of ABC transporters in the PA fraction. Substrate specificity of euryarchaeal ABC transporters indicated that Euryarchaea mainly use ABC transporters for amino acid and metal compounds acquisition (fig. S3B). Euryarchaea express high amounts of transcripts for amino acid transporters (*25*). Also, they have been identified as important protein degraders in the bathypelagic (*36*). Our proteomic analyses corroborate their role in particulate protein assimilation and remineralization. Recently, it has been suggested that zooplankton contribute ~30% of the particulate proteins in the deep sea (*26*) and therefore might be one of the major protein sources for Euryarchaea.

Substrate specificity analysis showed that ABC transporters mainly target amino acids, metal compounds (Fe, Zn, Mn, Mo, Cu, and Co), phosphorus (containing both organic phosphonate and inorganic phosphate), polyamine, sugars, and urea (Fig. 3B and dataset S3). These substrates specificity is consistent with a previous report (*15*). Changes in substrate preference of ABC transporters were obvious between size fractions in the metaproteome (Fig. 3, B and C). For example, the relative abundance of ABC transporters targeting amino acids was higher in the FL (23% to 54%; dataset S3) than the PA fraction, but ABC transporters of phosphorus (35 to 39%) and polyamine (6 to 17%) were relatively high in the PA fraction particularly in the bathypelagic (Fig. 3B). This change might be related to the taxonomic variation in ABC transporters between the FL and PA fraction (Fig. 3A).

### Taxonomic origin and substrate specificity of prokaryotic TBDTs remain relatively constant throughout the water column

For TBDTs, the taxonomic affiliations were mostly related to Gammaproteobacteria (27 to 41%), Bacteroidetes (7 to 22%), and Alphaproteobacteria (3 to 35%) irrespective of the size fraction and the “omics” level, but the contribution of each taxon varied in different omics samples (Fig. 3C). For example, while Gammaproteobacteria were the major contributor to TBDTs in all omics samples, Bacteroidetes TBDTs exhibited a high relative abundance in the metagenome (14 to 22%) and metatranscriptome (8 to 17%). Their protein abundance, however, was less prominent (9 to 17% in the FL fraction and 6 to 11% in the PA fraction; Fig. 3C and dataset S2) (*20*). In contrast, Alphaproteobacteria exhibited a high contribution to the TBDT gene pool in the bathypelagic metagenome (35 ± 15%), and the corresponding protein abundance in the metaproteome accounted for 10 to 17% in the FL fraction and 10 to 20% in the PA fraction (Fig. 3C and dataset S2).

In the metaproteome, ABC transporters (Fig. 3A) showed pronounced differences between size fractions and the contribution of Cyanobacteria, and Euryarchaea exhibited opposite trends in the PA fraction throughout the water column. In contrast, the taxonomic composition of TBDTs did not vary between size fractions or depth layers as reflected in all omics datasets (Fig. 3C).

TBDTs were used for chito-oligosaccharides (DOM_Cluster_3090), arabinose (DOM_Cluster_427), digested proteins, starch (DOM_Cluster_720), vitamin B_12_ (SiderophoresVitamin_Cluster_973), and iron containing siderophore and thiamin (SiderophoresVitamin_Cluster_410; Fig. 3D and table S2). The substrate specificity of TBDTs in the FL fraction remained relatively constant throughout water column but became variable in the PA fraction especially in the meso- and bathypelagic layers (Fig. 3D).

### Multi-omics analysis reveals a dynamic prokaryotic response to organic matter supply

In the deep-sea metatranscriptome distinct patterns were found in the taxonomic composition of ABC transporters and TBDTs (Fig. 3A and dataset S2). Verrucomicrobiota (7.8 ± 1.9%) and several unclassified bacteria (22 ± 6%) contributed substantially to the ABC transporter transcripts in the bathypelagic (Fig. 3A). High expression of transcripts encoding ABC transporters (7.7 ± 3.4%) and TBDTs (17 ± 6%) in Bacteroidetes was also detected. The low TBDT/ABC ratio at the transcript level in the bathypelagic (Fig. 1C) might be biased due to changes in the taxonomic composition of active communities in the deep sea. The depressurization of the deep-sea samples upon hoisting on board the research vessel substantially changes the metabolic activity of pressure-sensitive bacteria (*21*). Leucine incorporation rates of Bacteroidetes are up to 100 times higher under atmospheric than under the hydrostatic pressure conditions of the deep sea (*22*). Metatranscriptomic analysis of marine prokaryotic communities revealed distinct patterns in their response to environmental change in the global epi- and mesopelagic ocean (*11*, *28*), but metatranscriptomic samples from the deep ocean might be vulnerable to pressure changes (*22*) and the fairly long time elapsing between sampling at depth and lastly fixing the samples on board of the ship (*32*). Considering this variability in metatranscriptomic analysis particularly in deep-sea samples, results solely from metatranscriptomic analyses need to be cautiously interpreted. Hence, a combination of multi-omics tools might provide valuable additional information on prokaryotic metabolism in the deep ocean.

Substrate specificity did not change throughout the water column in the metagenome and the metatranscriptome (except for the bathypelagic metatranscriptome) (Fig. 3, B and D). In the metaproteome, depth-related and/or size-fractionated variability was found in the relative abundance of transporters (both ABC transporters and TBDTs) targeting different substrates (Fig. 3, B and D). This might be related to differences in the POM flux in oceanic waters (*24*, *37*) as the metaproteomes were collected from several and contrasting oceanic regions (dataset S1). Despite the clear difference in the relative abundance of proteins and corresponding genes/transcripts revealed by different omics analyses, the substrate categoryies for both ABC transporters and TBDTs remained fairly constant throughout water column in all omics samples (Fig. 3, B and D). The consistency of substrate specificity has been previously reported for the FL and PA prokaryotic communities (*8*, *9*). This is because POM and the DOM solubilized from the POM, rather than refractory DOM, in the ambient deep-water support the metabolic activity of the deep-sea microbiota (*19*). This observation is also consistent with the pattern of extracellular enzyme production throughout water column, where the function of extracellular enzymes also remains fairly constant with depth (*20*). It is noticeable that we used 0.8 μm as the cutoff for all PA prokaryotic communities, which may potentially lead to biases if FL bacteria are of large-cell size. Serial filtration with decreasing filter pore sizes (i.e., 0.2 to 0.8 μm, 0.8 to 3 μm, 3 to 20 μm, and 20 to 200 μm) will improve our understanding on the substrate acquisition patterns of prokaryotes colonizing the particle continuums.

Nevertheless, metaproteomic analysis of the substrate specificity indicated that ABC transporters were mainly responsible for amino acid uptake (Fig. 3B), while carbohydrate uptake was largely mediated by TBDTs (Fig. 3D). Taxonomic variation between size fractions in substrate preference revealed distinct roles of different prokaryotes in marine organic carbon and nitrogen cycling.

### Substrate uptake exhibits distinct patterns in major taxonomic groups

Metaproteomic analysis of the substrate specificity indicated that ABC transporters are mainly responsible for amino acid uptake (Fig. 3, A and C), but carbohydrate uptake is largely maintained by TBDTs (Fig. 3, B and D). Such a substrate preference reveals distinct roles of different prokaryotic taxa in marine organic carbon and nitrogen cycling. However, taxonomic classification remains vague because Alphaproteobacteria and Gammaproteobacteria contain diverse taxonomic groups.

**Fig. 4.**
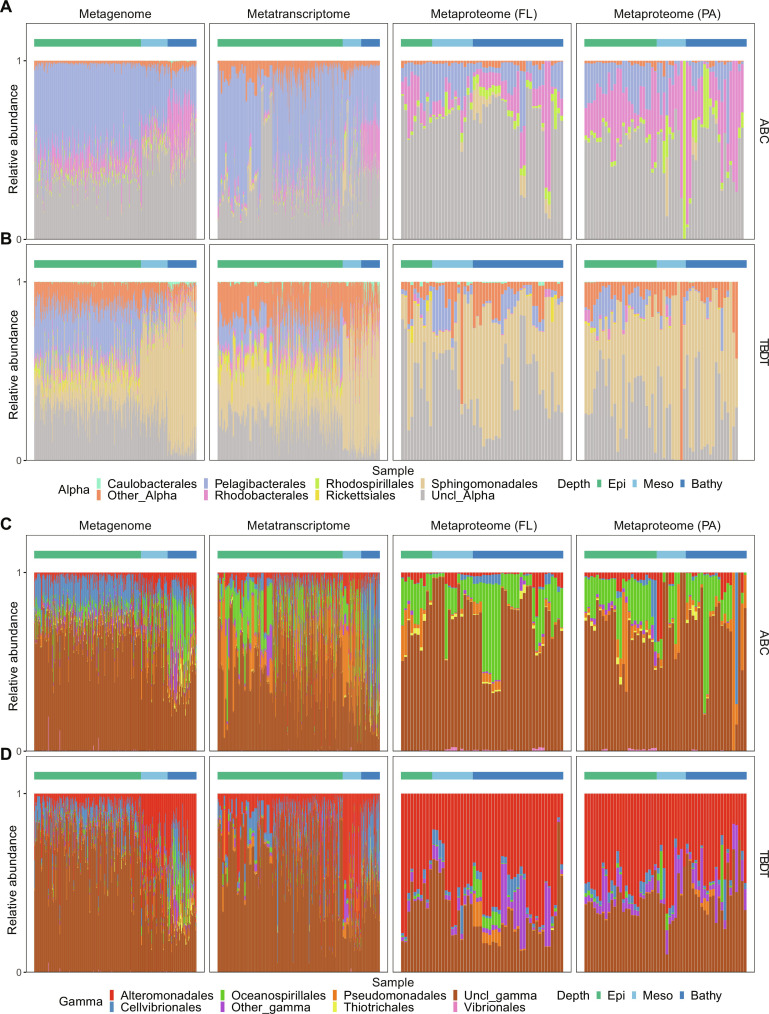
Distribution of ABC transporters and TBDTs in Alphaproteobacteria and Gammaproteobacteria reflected by metagenomic, metatranscriptomic, and metaproteomic analysis. (**A** and **B**) Order level classification of ABC transporters and TBDTs in Alphaproteobacteria. (**C** and **D**) Order level classification of ABC transporters and TBDTs in Gammaproteobacteria. The *x* axis represents samples from different stations, and the depth layers of the sample are indicated using the color bar on the top. The horizontal color bar refers to sampling depth as defined in Fig. 1C.

We further examined the transporter composition of three major taxonomic groups, i.e., Alphaproteobacteria, Gammaproteobacteria, and Bacteroidetes (Fig. 4 and figs. S4 and S5). Within the Alphaproteobacteria, Pelagibacterales (SAR11) maintained the largest gene (49 ± 8%) and transcript (51 ± 19%) pool of ABC transporters in the epipelagic, while Rhodobacterales-affiliated ABC transporter genes (24 ± 12%) and transcripts (24 ± 6%) were more abundant in the bathypelagic (Fig. 4A and dataset S4). Moreover, the protein abundance of ABC transporters in Rhodobacterales was much higher in the PA (32 ± 25%) than the FL fraction (14 ± 16%; Fig. 4A and dataset S4). In contrast, Sphingomonadales contributed prominently to alphaproteobacterial TBDTs particularly in the meso- and bathypelagic layers (48 to 78% in the metagenome, 59 to 64% in the metatranscriptome, and 25 to 65% in the metaproteome of both the FL and PA fraction; Fig. 4B and dataset S4). The overexpression of TBDTs in Sphingomonadales has been reported for plant-associated microbial communities (*38*) but not in the marine environment. Similar to Euryarchaea, Sphingomonadales also exhibited a low cell abundance (fig. S4A) but a high protein abundance (fig. S4A). Thus, considering the important role of TBDTs in organic carbon uptake, Sphingomonadales have not received adequate attention yet probably due to their low abundance (fig. S4A), particularly in its role in organic matter uptake in the deep sea. In Gammaproteobacteria, Oceanosprillales mainly used ABC transporters, while Alteromonadales used TBDTs (Fig. 4, C and D). Alteromonadales-affiliated TBDTs constituted 38 to 56% of gammaproteobacterial TBDTs in the metaproteome, but the relative abundance of corresponding genes/transcripts was slightly lower in the metagenome (10 to 38%) and metatranscriptome (15 to 54%; Fig. 4D and dataset S4).

**Fig. 5.**
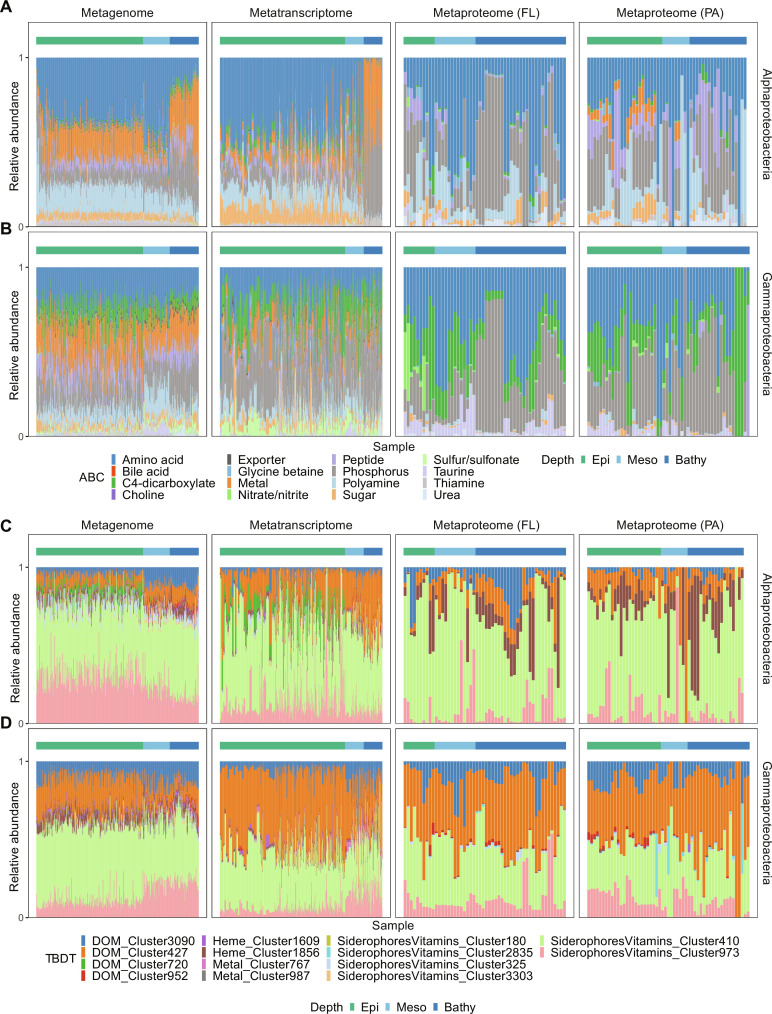
Substrate specificity of ABC transporters and TBDTs in Alphaproteobacteria and Gammaproteobacteria reflected by metagenomic, metatranscriptomic, and metaproteomic analysis. (**A** and **B**) Substrate specificity of ABC transporters in Alphaproteobacteria and Gammaproteobacteria. (**C** and **D**) Substrate specificity of TBDTs in Alphaproteobacteria and Gammaproteobacteria. The *x*-axis represents samples from different station, and the depth layers of the sample is indicated using the color bar on the top. The horizontal color bar refers to sampling depth as defined in Fig. 1C.

By linking substrate specificity to transporters in different taxonomic groups (i.e., Alphaproteobacteria versus Gammaproteobacteria), we found clear differences in substrate acquisition patterns (Fig. 5). While both Alphaproteobacteria and Gammaproteobacteria have ABC transporters for amino acid and phosphorus uptake (Fig. 5, A and B), Alphaproteobacteria also used ABC transporters for polyamines (Fig. 5A). Taurine and C4-dicarboxylate, as carbon and energy sources for marine prokaryotes, were taken up by Gammproteobacteria such as Oceanospirillales using ABC transporters (Figs. 4C and 5B. Polyamines are nitrogen-rich phytoplankton exudates (*39*) and directly accessible to bacteria such as SAR11 (*40*, *41*). Our data showed that SAR11 dominated the ABC transporter pool in the epipelagic waters (Fig. 4A). In SAR11, the gene, transcript, and protein abundance of polyamine transporters were also high (Fig. 5A).

Major differences in substrate preference in TBDTs between Alphaproteobacteria and Gammaproteobacteria were also found. As Sphingomonadales was the major group within the Alphaproteobacteria using TBDTs (Fig. 4B), the substrate affinity of alphaproteobacterial TBDTs was mainly shaped by this group (Fig. 5B and fig. S4B). In both the FL and PA fraction of the metaproteome, about 80% of alphaproteobacterial TBDTs targeted heme (Heme_Cluster_1856), siderophore (SiderophoreVitamin_Cluster_410), and vitamin B_12_ (SiderophoreVitamin_Cluster_973, Fig. 5C and table S2). In contrast, 50% of gammaproteobacterial TBDTs targeted siderophores (SiderophoreVitamin_Cluster_410) and vitamin B_12_ (SiderophoreVitamin_Cluster_973; Fig. 5D and table S2), and the heme-related TBDTs were barely detected in Gammaproteobacteria. Heme is an iron-containing cofactor used by phytoplankton for photosynthesis, and its concentration is used as proxy for iron flux from POM into the ambient water column (*42*, *43*). The prevalence of heme-related TBDTs in Alphaproteobacteria (mainly Sphingomonadales) suggests a unique iron acquisition pattern from sinking algae. In contrast, the high relative abundance of carbohydrate-related TBDTs in Gammaproteobacteria (~50%) suggests extensive activity in organic carbon assimilation and remineralization by this taxonomic group. Hence, the polysaccharides and metal chelate such as iron originating from sinking algae are probably cleaved by different taxonomic groups. It is noticeable that, although the relative abundance of DOM related TBDTs (DOM_Cluster_3090 and DOM_Cluster_427) varied between Alphaproteobacteria (10 to 15%; Fig. 5C) and Gammaproteobacteria (~50%; Fig. 5D), TBDTs targeting arabinose (DOM_Cluster_427) seem to be important for both Alphaproteobacteria and Gammaproteobacteria. Arabinose is a cell wall monosaccharide of diatoms and constitutes 2 to 5% of sinking and suspended particles in the deep ocean (*44*, *45*). Decaying phytoplankton blooms could lead high flux rates of arabinose from surface to the deep ocean (*44*), which may fuel deep-sea prokaryotes such as Sphingomonadales and Alteromonadales. In addition to arabinose, other monosaccharides such as xylose and xylan and polysaccharides such as chitin and pectin were also assimilated by Alphaproteobacteria and Gammaproteobacteria using TBDTs (DOM_Cluster_3090).

Compared to Alphaproteobacteria and Gammaproteobacteria, Bacteroidetes exhibited distinct substrate acquisition patterns (fig. S5). Although Bacteroidetes had the potential to take up amino acids, peptides, and sugars using ABC transporters, the expressed ABC transporter proteins in Bacteroidetes mainly targeted phosphorus (fig. S5A). Yet, Bacteroidetes assimilated bulk organic nitrogen (cleaved proteins) and organic carbon (starch) using TBDTs (DOM_Cluster_720, fig. S5B). Even for siderophore and vitamin (thiamin), Bacteroidetes used various types of TBDTs (SiderophoreVitamin_Cluster_180; fig. S5B). This substrate assimilation pattern differed markedly from both Alphaproteobacteria and Gammaproteobacteria. The only common feature in the substrate assimilation of Bacteroidetes, Alphaproteobacteria, and Gammaproteobacteria is that they all use ABC transporters for phosphorus and TBDTs (SiderophoreVitamin_Cluster_180) for vitamin B_12_ uptake (Figs. 4 and 5, and fig. S5). Both phosphorus and vitamin B_12_ are important for DNA metabolism (*46*–*48*), which is further related to cell reproduction.

The contrasting patterns of substrate specificity of transporters among taxonomic groups revealed prokaryotic preferences to specific types of monomers and indicated their role in the organic matter cycle. For example, ABC transporters were mainly used by SAR11 and Rhodobacterales for organic nitrogen (amino acid) uptake, but TBDTs were responsible for organic carbon uptake in Alteromonadales. However, Bacteroidetes used TBDTs for both organic carbon and nitrogen (DOM_Cluster_720; fig. S5B). This assimilation pattern is also partially linked to their capability of extracellular enzyme secretion (*20*). TBDT users such as Sphingomonadales and Alteromonadales secrete both carbohydrate active enzymes (CAZymes) and peptidases, while Rhodobacterales secrete peptidases and use ABC transporters to take up amino acids. Thus, the cleavage pattern is closely linked to the presence of transporters, together facilitating a particle associated lifestyle of prokaryotes especially in the deep ocean.

### Transporter expression supports different lifestyles of deep-sea prokaryotes

The ratio of TBDT/ABC transporters in the metagenome and the metaproteome revealed that deep-sea prokaryotes tended to use TBDTs in the PA fraction in the deep ocean (Fig. 1, C and F). Taxonomic analysis showed that Alteromonadales, Bacteroidetes, and Sphingomonadales were major contributors of TBDTs in the metaproteome (both PA and FL fraction) in the deep sea. Recent study shows that Alteromonadales, Bacteroidetes, and Sphingomonadales are among most active prokaryotes in the deep sea but exhibits distinct lifestyle (*49*). Alteromonadales and Bacteroidetes are major taxa of PA heterotrophic bacteria in the deep ocean (*24*). Alteromonadales and Bacteroidetes cells are characterized by high cell-specific leucine incorporation rates (fig. S6), suggesting an active role in deep-sea carbon cycle. Both of them are inhibited by high hydrostatic pressure, but their metabolic response to the high hydrostatic pressure differs from each other (*22*). In contrast, Sphingomonadales, which contributes 50% of 16*S* ribosomal RNA transcripts in the FL fraction (*49*), also expressed substantial amounts of TBDTs in both the PA and FL fraction in our metaproteome (Fig. 4B). Sphingomonadales seems to be capable of assimilating organic matter solubilized from POM and DOM from the ambient water. How their metabolism is related to the lifestyle (FL versus PA) and the role of transporters (ABC transporter versus TBDT) in substrate uptake has not been determined yet. We first examined the protein expression profiles of Alteromonadales, Bacteroidetes, and Sphingomonadales in the FL and PA fraction throughout the water column using the approach of Pachiadaki *et al.* (*50*). We collected metagenomic-assembled genomes (MAGs) of Alteromonadales, Bacteroidetes, and Sphingomonadales from the global ocean (*51*, *52*) and constructed a nonredundant gene collection at the species level for each taxon. Using this gene collection as reference database for metaproteomic analysis, we found that the protein expression profiles (fig. S7) for these three taxa showed a clear clustering pattern between size fractions and depth layers. In Alteromonadales, TBDTs (TC.FEV.OM), flagella proteins (flgE), and microbial chemotaxis proteins (mcp) were among the most abundant proteins (>5%; fig. S7), suggesting a high tendency toward a PA lifestyle (*53*). In Bacteroidetes, proteins/enzymes involved in anti-oxidative stress (ahpC and peroxiredoxin) were much higher in the bathypelagic than in the epipelagic and mesopelagic waters (fig. S7). In the contrast, ahpC in Alteromonadales was less prominent, suggesting different adaptation mechanism to oxidative stress under high hydrostatic pressure conditions (*54*). The protein expression of Sphingomonadales exhibited a different profile than Alteromonadales or Bacteroidetes. Although the detection of superoxide dismutase (SOD2; fig. S7) in the Sphingomonadales proteome also suggested oxidative stress induced by high hydrostatic pressure, both ABC transporters (pstB/S) and TBDTs (TC.FEV.OM) were relatively high in Sphingomonadales (fig. S7). However, the tendency toward particles in Sphingomonadales or Bacteroidetes seemed limited because mobility-related proteins (flagella proteins and chemotaxis proteins) were low in relative abundance (fig. S7).

**Fig. 6.**
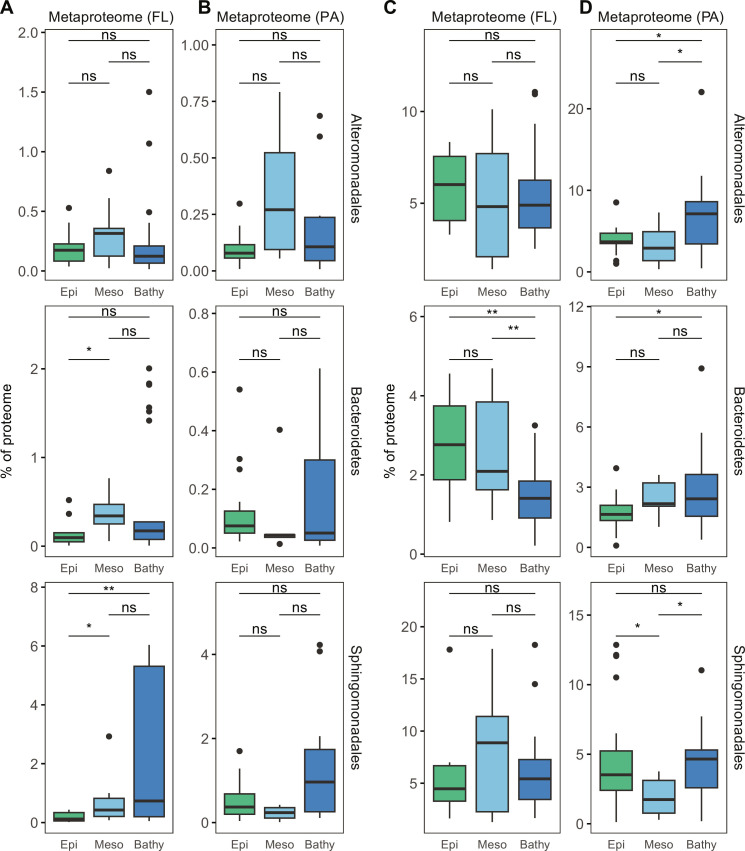
Relative abundance of ABC transporters and TBDTs in the proteome of Alteromonadales, Bacteroidetes, and Sphingomonadales. The protein expression of ABC transporters (**A** to **B**) and TBDTs (**C** to **D**) varied in Alteromonadales, Bacteroidetes, and Sphingomonadales between FL and PA fractions throughout the water column. Box shows median and IQR; whiskers show 1.5 × IQR of the lower and upper quartiles or range; outliers extend to the data range. Statistics are based on Wilcoxon test. *P < 0.05 and **P < 0.01.

We further compared the expression of transporter proteins in the proteome of Alteromonadales, Bacteroidetes, and Sphingomonadales. ABC transporters (Fig. 6A-B) constituted 0.1 to 0.7% of the proteome in Alteromonadales (0.1 to 0.3%; median = 0.17%), Bacteroidetes (0.1 to 0.5%, median = 0.34%), and Sphingomonadales (0.2 to 2%, median = 0.7%). TBDTs accounted 2 to 8% in the proteome of Alteromonadales (4 to 6%), Bacteroidetes (2 to 5%), and Sphingomonadales (2 to 8%; Fig. 6, C and D). The depth-related relative abundance of transporters significantly differed between these three taxa. In Alteromonadales, the protein abundance of TBDTs did not change with depth in the FL fraction but significantly increased toward deeper waters in the PA fraction (Fig. 6, C and D). In contrast, the protein abundance of TBDTs in Bacteroidetes decreased remarkably toward the bathypelagic waters in the FL fraction, while protein abundance of Bacteroidetes-affiliated TBDTs remained high in the PA fraction (Fig. 6C-D). In Sphingomonadales, the relative abundance of TBDTs in the deep-sea PA fraction was similar to that in the epipelagic layer. Yet, the overall abundance of TBDTs in the PA fraction (2 to 4%) was slightly (median-to-median comparison) lower than in the FL fraction (5 to 8%). Clear differences in substrate specificity between size fractions were also found in Sphingomonadales-affiliated TBDTs in the metaproteome especially in the deep ocean (Fig. 5C). In the PA fraction, TBDTs targeting heme (Heme_Cluster_1856) was significantly higher (*P* < 0.05, Wilcoxon test) relative abundance (27 ± 24%) than the FL fraction (15 ± 16%), while in the FL fraction, the relative abundance of TBDTs targeting various DOM (12 ± 11% of DOM_Cluster_3090 and 12 ± 8% of DOM_Cluster_427) was higher than that in the PA fraction (4 ± 3% of DOM_Cluster_3090 and 8 ± 4% of DOM_Cluster_427; *P* < 0.05, Wilcoxon test; Fig. 5C). This indicates Sphingomonadales can regulate the abundance of different types of TBDTs according to its lifestyle.

Compared to TBDTs, the relative abundance of ABC transporters remained fairly constant in the Alteromonadales and Bacteroidetes proteome throughout the water column regardless the size fraction (Fig. 6A). In Sphingomonadales, the relative abundance of ABC transporters was significantly higher (*P* < 0.05, Wilcoxon test) in the meso- and bathypelagic in the FL than in the PA fraction (Fig. 6A). Thus, the expression of transporter proteins (Fig. 6) together with the protein expression of each taxon (fig. S7) suggests that Sphingomonadales can use ABC transporters and TBDTs in both the FL and PA fraction in the deep ocean. The increased expression of ABC transporters (Fig. 6A) and the change in substrate specificity of TBDTs (Fig. 5C) in Sphingomonadales in the FL fraction in the deep ocean might support its high activity as recently reported (*49*). Alteromonadales tends to colonize particles (fig. S7) and assimilate organic substrates using TBDTs (Fig. 5D), Bacteroidetes can also benefit from POM supply to the deep ocean (Fig. 6D and fig. S5). However, the importance of Bacteroidetes in deep-sea organic matter cycling might decrease if detached from particles, as reflected by the decrease of TBDTs in the FL fraction in the bathypelagic realm (Fig. 6C).

Variations in prokaryotic transporter expression in marine ecosystems indicate different roles of prokaryotic taxa in marine organic matter cycling (*14*, *15*). While the genetic profile indicates long-term adaptive and potential mechanisms of prokaryotes, gene expression into mRNA and proteins ultimately determines the prokaryotic response to organic matter supply and availability (*8*–*11*, *13*). The decreasing bioavailability of DOM with depth in the water column (*55*) renders POM utilization the most important source of fresh organic carbon and nitrogen for dark ocean prokaryotes.

Applying different omics analyses revealed distinct profiles in the potential, response, and expression of marine prokaryotic transporters. Linking the taxonomic origin and substrate specificity to different types of transporters, a clear niche separation in prokaryote-mediated organic matter cycles was found. By examining protein expression levels in the major prokaryotic groups, we found that the lifestyles of deep-sea prokaryotes are closely related to their metabolism and transporter expressions, which lead to a differentiation of their roles in deep-sea organic matter cycling. In summary, fine-scale differentiation of transporters at the gene, transcript, and protein level allows unveiling the ecological niches of prokaryotic taxa.

## MATERIALS AND METHODS

### Metaproteomic sampling

The sampling protocol was described in previous publications (*20*, *26*). Briefly, about 100 to 400 liters of seawater were sequentially filtered through 0.8- and 0.2-μm pore size polycarbonate membranes (142 mm in diameter) at 22 stations in the Pacific, Atlantic, and Southern Ocean using air pumps operated at a pressure between 1.5 and 2.0 bar. Two filtration manifolds (Sartorius) were used to speed up the filtration process, generally performed within 1.5 to 2 hours. The filters were immediately frozen in liquid nitrogen and stored at −80°C until extraction. Protein extraction was performed in the laboratory using lysis buffer containing 7 M urea, 2 M thiourea, 1% dithiothreitol, 2% CHAPS, and protease inhibitor cocktail. The mixture was homogenized with bead beating and sonicated at high power with 10-s pulses for 10 min. The supernatant from the slurry and the concentrated filtrates were further concentrated to 250 μl with a 3000-Da Amicon Ultra-15 Centrifugal Filter Unit (Millipore). The protein fraction was precipitated with cold ethanol overnight at −20°C and resuspended with 7 M urea and 2 M thiourea. The protein pellet of each sample was digested using the filter-aided in-solution trypsin digestion method (1:100, w/w) (*56*). The peptides were sequenced on a Q-Exactive Hybrid Quadrupole-Orbitrap Mass Spectrometer (Thermo Fisher Scientific) after desalting. The tryptic peptide pellets were dissolved in 4% (v/v) acetonitrile and 0.1% (v/v) formic acid. Samples (at least two replicates) were loaded on C18 reverse-phase columns (Thermo Fisher Scientific, EASY-Spray 500 mm, 2-μm particle size). Separation was achieved with a 90-min gradient from 98% solution A [0.1% formic acid in high purity water (Milli-Q)] and 2% solution B (90% acetonitrile (ACN) and 0.1% formic acid) at 0 min to 40% solution B (90% ACN and 0.1% formic acid) at 90 min with a flow rate of 300 nl min^−1^. Nano electrospray ionization mass spectrometry (ESI-MS)/MS measurements were performed on Orbitrap QExactive (Thermo Fisher Scientific, Bremen, Germany) with the following settings: full scan range 350 to 1800 mass/charge ratio and resolution of 120,000 max. Twenty MS2 scans (activation-type collision-induced dissociation), repeat count of 1, repeat duration of 30 s, exclusion list size of 500, exclusion duration of 30 s, charge state screening enabled with the rejection of unassigned and +1 charge states, and minimum signal threshold of 500. The mass spectrometry proteomics data were submitted to the ProteomeXchange Consortium via the PRIDE (*57*) partner repository with the dataset identifier PXD034421.

### Acquisition of gene catalogs for the marine prokaryotic community

Metagenomic reads downloaded from the National Center for Biotechnology Information (NCBI) website together with in-house sequencing results were used to construct a prokaryotic gene catalog (*26*). Reads from the metagenomic dataset were assembled individually using MEGAHIT (1.1.2) (*58*) with default settings. Putative genes were then predicted on contigs longer than 200 base pair using Prodigal (2.6.3) (*59*) under metagenome mode (-p meta) and further clustered at 90% similarity (-c 0.9 -G 0 -aS 0.9) using CD-HIT (4.6.8) (*60*) to construct the prokaryotic gene database for downstream metaproteomic analysis. Metagenomic (*8*, *20*, *29*, *52*) and metatranscriptomic (*28*) reads (dataset S1) were mapped back to the gene catalog using Burrows-Wheeler Aligner (*61*) with a threshold of minimum identity >95%. Gene/transcript abundance was calculated as follows: gene/transcripts abundance = mapped reads/length/sum (mapped reads/length) × 10^6^. Analysis of phylogenetic mOTUs (*33*) was performed for both the metagenomics and metatranscriptomic dataset to cover the prokaryotic community for both total and active ones, respectively.

### Prokayotic transporter gene catalog construction.

The predicted genes were annotated with both the Kyoto Encyclopedia of Genes and Genomes (KEGG) (*62*) and eggNOG database (*63*) with priority given to eggNOG annotation results. Genes with an annotation result including keywords “ABC transporter” or “TonB dependent receptor” were kept as positive hits and further checked with the Transporter Classification Database (TCDB, 3.A.1 for ABC transporters and 2.C.1 for TBDTs) (*64*). Substrate specificity of ABC transporters was retrieved from eggnog/KEGG annotations. For TBDTs, substrate prediction was made by searching (blastp) TBDT such as sequences against a curated TBDT sequence database of pure cultures (*14*) using a threshold of identity ≥ 30%. Protein sequences of TBDTs targeting different substrates can be found in dataset S5.

### Proteomic annotation and analysis

Because of the large size of the gene catalogs, a two-step database searching method was used (*65*). Briefly, the MS/MS spectra of proteomic samples were pooled and searched using SEQUEST-HT (*66*) engines against proteins in the databases of eukaryotes, prokaryotes and viruses with a false discovery rate (FDR) of 10% (*26*). Sequences identified in this step were exported as a “trimmed” database. The trimmed database was combined with transporter protein sequences for the second search where the proteomic samples were analyzed separately. In this step, FDR was set to 1% for protein selection, and the Xcorr threshold was established at 1 per charge (2 for +2 ions 3 for +3 ions, etc.). The variable modifications were set to acetylation of the N terminus and methionine oxidation, with a mass tolerance of 10 parts per million for the parent ion and 0.8 Da for the fragment ion. The number of missed and nonspecific cleavages permitted was two, and only dynamic modifications were used. Percolator in Proteome Discoverer 2.1 (Thermo Fisher Scientific) was used for validation. A minimum of two peptides and one unique peptide were required for protein identification. Taxonomic affiliation of sequences was determined using the lowest common ancestor (diamond blastp --top 10 --sallseqid -outfmt 102) algorithm adapted from DIAMOND (v2.0.9) (*67*) blast by searching against the nonredundant (NR) database downloaded from NCBI in March 2022. The top 10% hits with an *e* value <1 × 10^−5^ were used for taxon determination (--top 10). Protein quantification was conducted with a normalized area abundance factor (NAAF) representing a chromatographic peak area–based label-free quantitative method (*68*), where NAAF = peak area/protein length/sum (peak area/protein length). Only the peak area from a unique peptide (a peptide not been shared by proteins or protein groups) and razor peptide (a peptide assigned to the protein with the highest number of unique peptides identified but with the shortest length) was used for the quantitation.

To evaluate the protein expression profile in Alteromonadales, Bacteroidetes, and Sphingomonadales between different size fractions in the water column, the trimmed database was further combined with protein sequences derived from MAGs of Alteromonadales, Bacteroidetes, and Sphingomonadales. Those MAGs were obtained from previous publications (*22*, *51*, *69*) and are available in Figshare (https://figshare.com/s/efdafb98a9c2ff8b57ed).

### Determination of cell-specific leucine uptake rates

Cell-specific leucine uptake rates were measured as described in a previous study (*22*). Briefly, the size of the silver grain halo around each 4′,6-diamidino-2-phenylindole–positive cell was measured using Axio Vision SE64 Re4.9 (Carl Zeiss) following microautoradiography performed on catalysed reporter deposition (CARD)–fluorescence in situ hybridization (FISH) processed samples (MICRO-CARD-FISH). The halo areas were converted to leucine incorporation rates in mole per day with an equation obtained from correlating the total area of the halos with the bulk leucine incorporation rates (*70*).

### Statistical analysis and visualization

All the statistics and figures were done using specific packages in R (www.r-project.org/). Vegan (*71*), ggplot2 (*72*), and pheatmap (*73*) were used for ordination, diversity calculation, and visualization, respectively.
