# Supplementary material for: Substrate uptake patterns shape niche separation in marine prokaryotic microbiome
Source: Sci Adv. 2024 May 15;10(20):eadn5143. doi: 10.1126/sciadv.adn5143 (PMC11095472; doi:10.1126/sciadv.adn5143)
Supplement: Supplementary file 1 — Figs. S1 to S7 Tables S1 and S2 Legends for dataset S1 to S5 [file sciadv.adn5143_sm.pdf]

Science Advances
AAAS

Supplementary Materials for

Substrate uptake patterns shape niche separation in marine prokaryotic microbiome

Zihao Zhao *et al.*

Corresponding author: Zihao Zhao, zihao.zhao@univie.ac.at; Gerhard J. Herndl, gerhard.herndl@univie.ac.at

Sci. Adv. **10**, eadn5143 (2024)
DOI: 10.1126/sciadv.adn5143

The PDF file includes:

Figs. S1 to S7
Tables S1 and S2
Legends for dataset S1 to S5

Other Supplementary Material for this manuscript includes the following:

Dataset S1 to S5

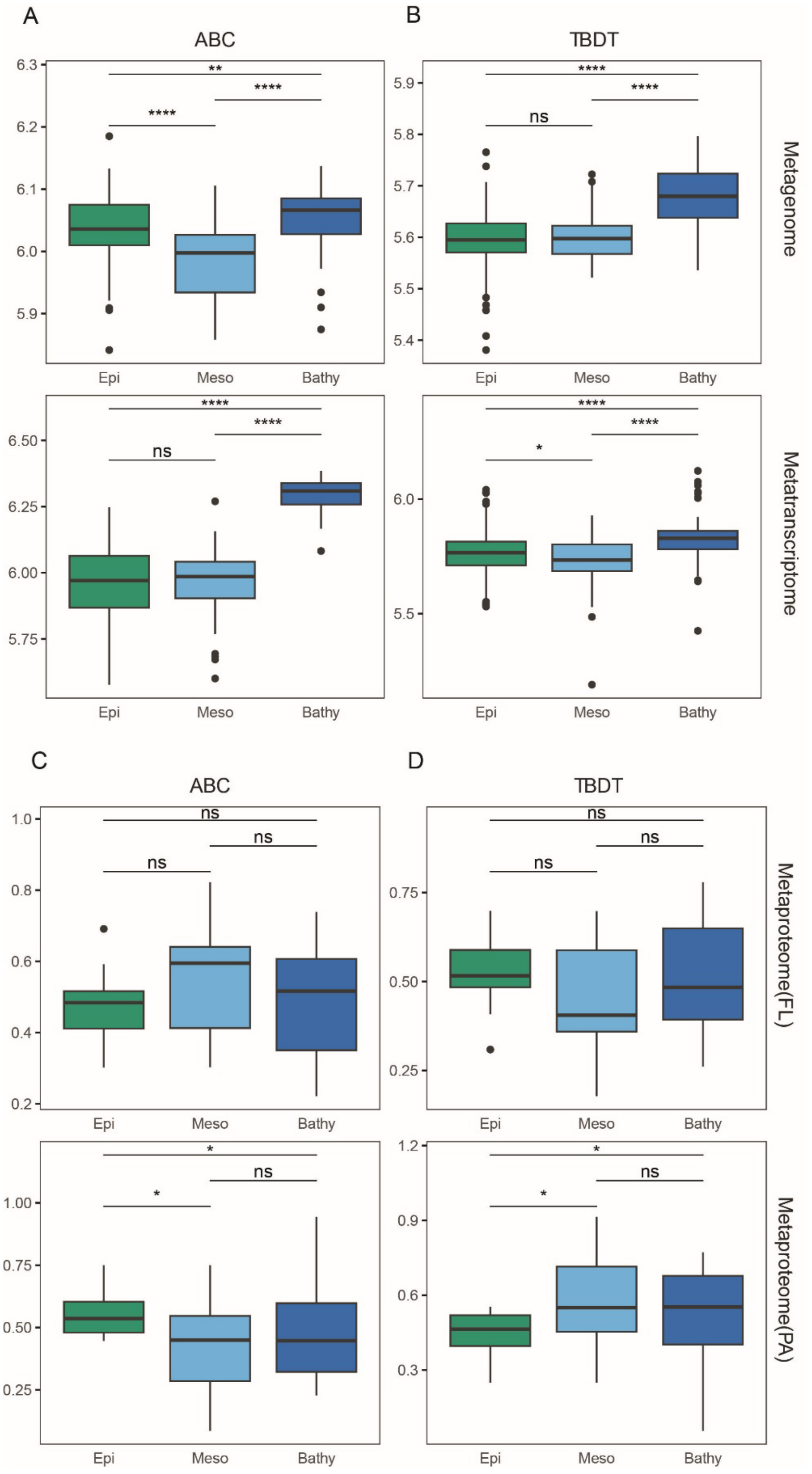

fig. S1 Sequence analysis of genes/transcripts encoding ABC transporters and TBDTs. A-B, gene and transcript abundance of ABC transporters and TBDTs in the metagenomic and metatranscriptomic dataset. **C-D,** protein abundance of ABC transporters and TBDTs in free-living (FL) and particle attached (PA) metaproteomes. The gene, transcript and protein abundances were log10 transformed. Box shows median and interquartile range (IQR); whiskers show 1.5 × IQR of the lower and upper quartiles or range; outliers extend to the data range. Statistics are based on Wilcoxon test. *P<0.05, **P<0.01, ***P<0.001, ****P<0.0001, ns, not significant.

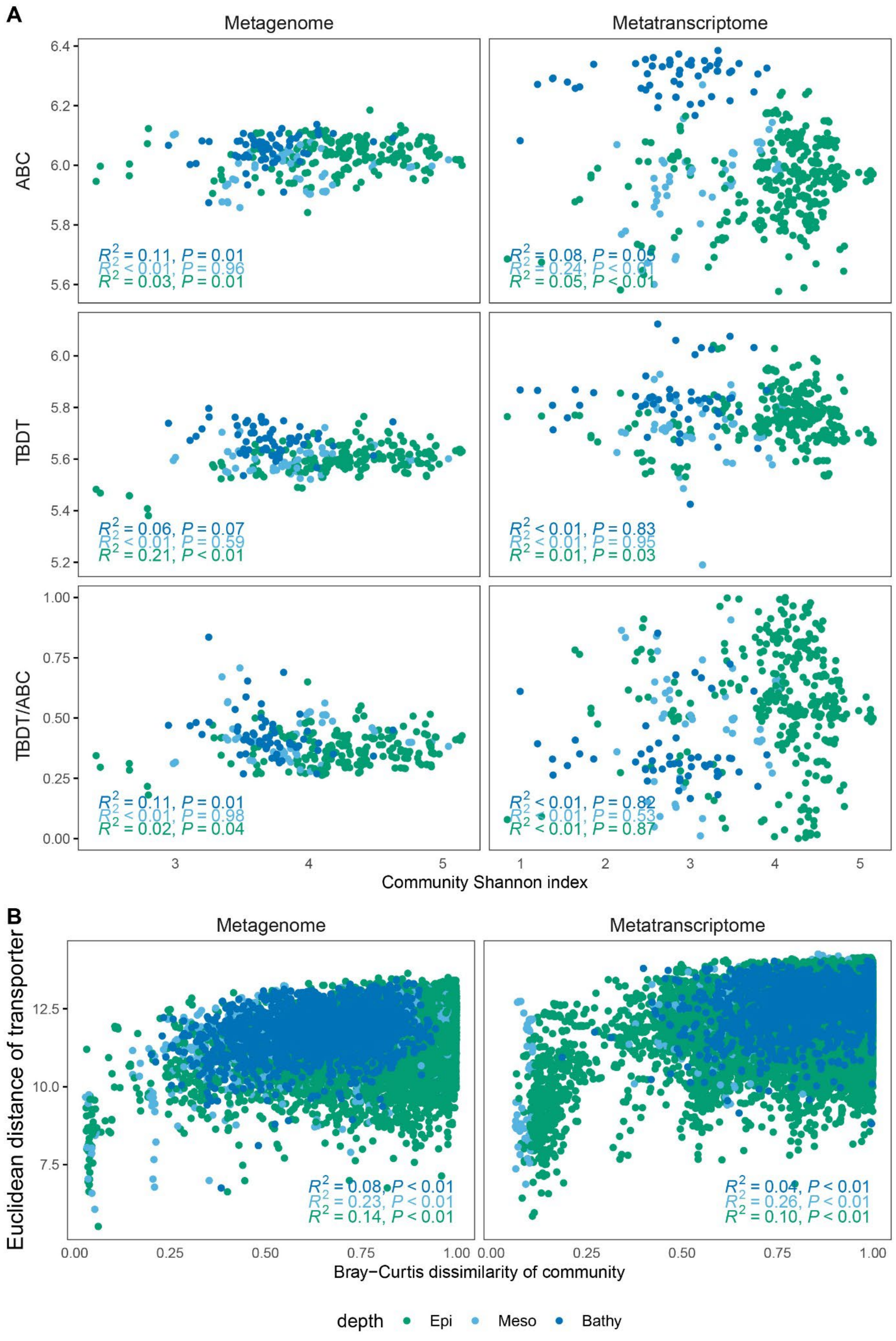

A
Metagenome
Metatranscriptome
ABC
TBDT
TBDT/ABC
R^2 = 0.11, P = 0.01
R^2 < 0.01, P = 0.96
R^2 = 0.03, P = 0.01
R^2 = 0.08, P = 0.05
R^2 = 0.24, P < 0.01
R^2 = 0.05, P < 0.01
R^2 = 0.06, P = 0.07
R^2 < 0.01, P = 0.59
R^2 = 0.21, P < 0.01
R^2 < 0.01, P = 0.83
R^2 < 0.01, P = 0.95
R^2 = 0.01, P = 0.03
R^2 = 0.11, P = 0.01
R^2 < 0.01, P = 0.98
R^2 = 0.02, P = 0.04
R^2 < 0.01, P = 0.82
R^2 < 0.01, P = 0.53
R^2 < 0.01, P = 0.87
Community Shannon index
B
Metagenome
Metatranscriptome
Euclidean distance of transporter
R^2 = 0.08, P < 0.01
R^2 = 0.23, P < 0.01
R^2 = 0.14, P < 0.01
R^2 = 0.04, P < 0.01
R^2 = 0.26, P < 0.01
R^2 = 0.10, P < 0.01
Bray−Curtis dissimilarity of community
depth
Epi
Meso
Bathy

fig. S2 Correlations between transporter profiles and community structure. A, relationship between community α-diversity (Shannon index) and transporter gene/transcript abundance and corresponding ratio**. B,** relationship between community variance (Bray-Curtis dissimilarity) and transporter composition (Euclidean distance of gene/transcript abundance and corresponding ratio)

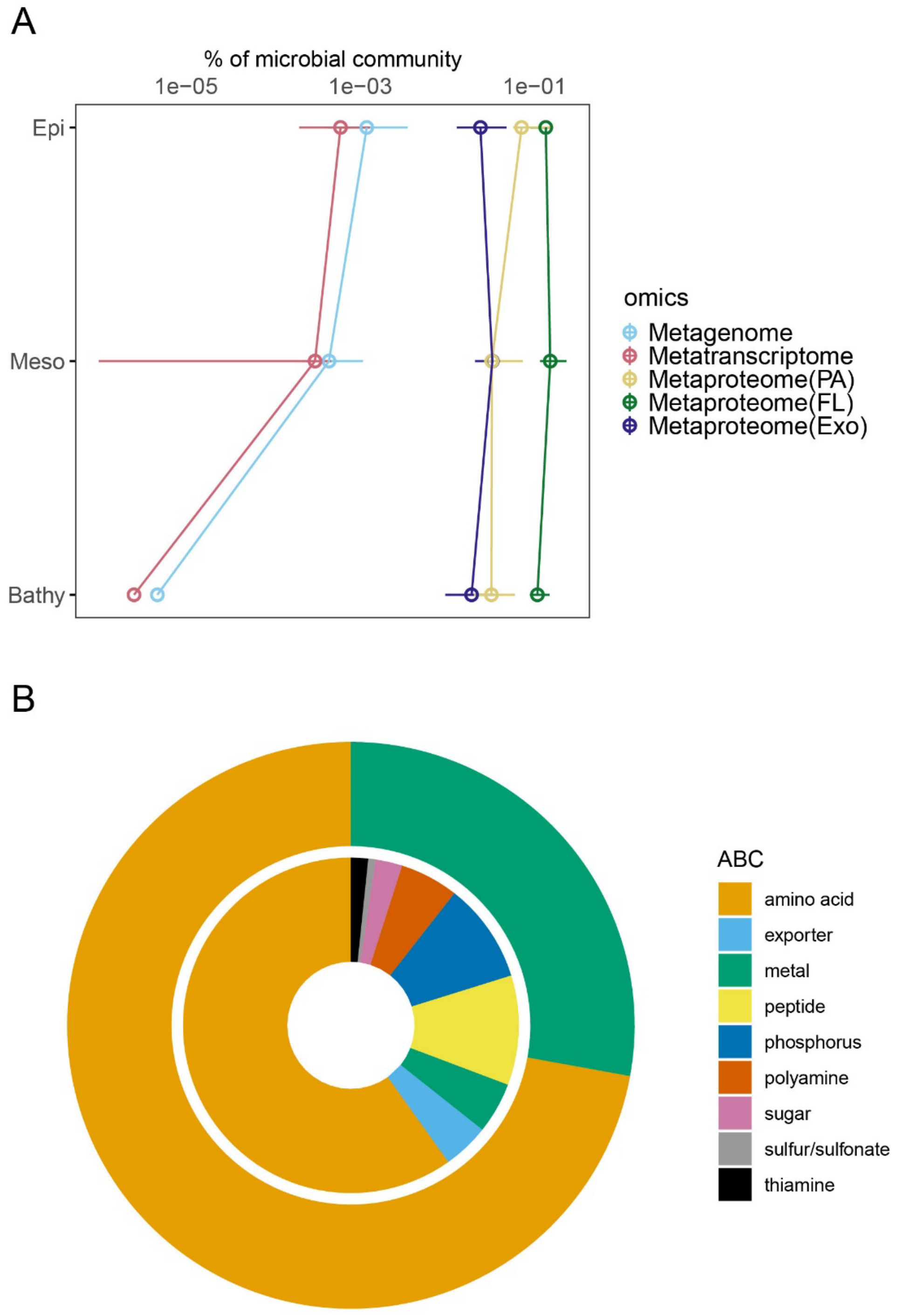

fig. S3 Contribution of Euryarchaea to ABC transporters. A, relative abundance of Euryarchaea in the metagenomic, metatranscriptomic and metaproteomic dataset. **B,** substrate specificity of gene (inner ring) and protein (outer ring) of euryarchaeal ABC transporters. PA, >0.8µm. FL, 0.2-0.8µm. Exo, <0.2µm.

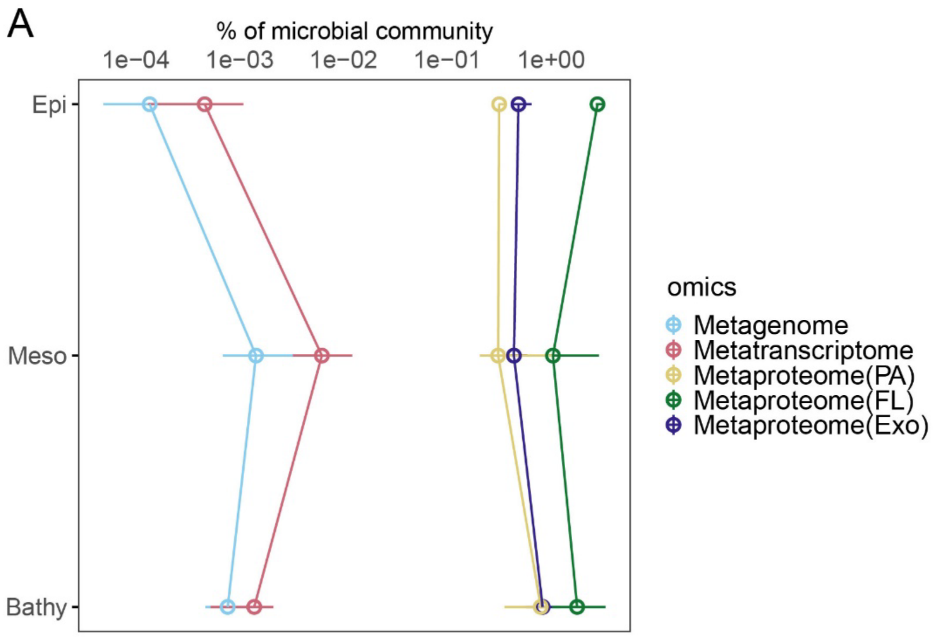

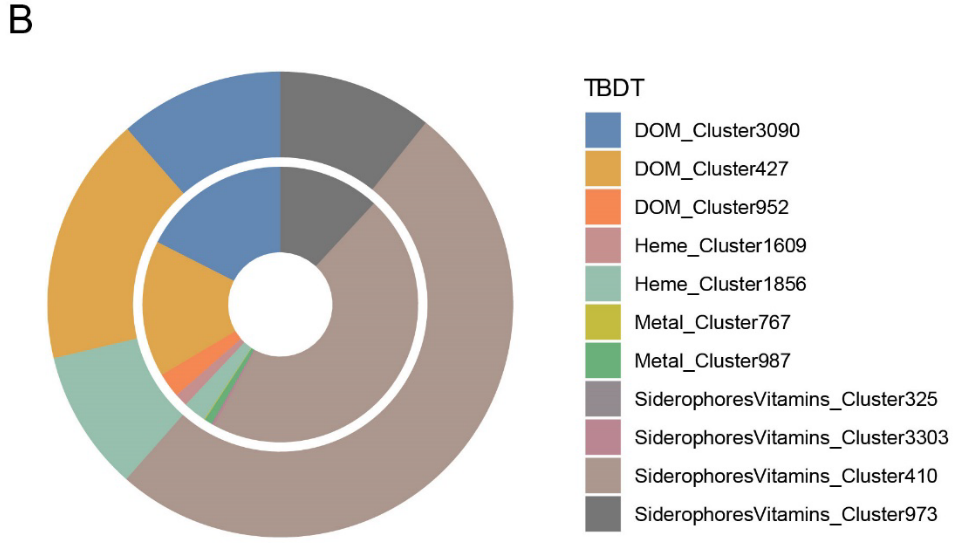

fig. S4 Contribution of Sphingomonadales to TBDTs. A, relative abundance of Sphingomonadales in the metagenomic, metatranscriptomic and metaproteomic dataset. **B,** substrate specificity of genes (inner ring) and proteins (outer ring) of Sphingomonadales-related TBDTs. PA, >0.8µm. FL, 0.2-0.8µm. Exo, <0.2µm.

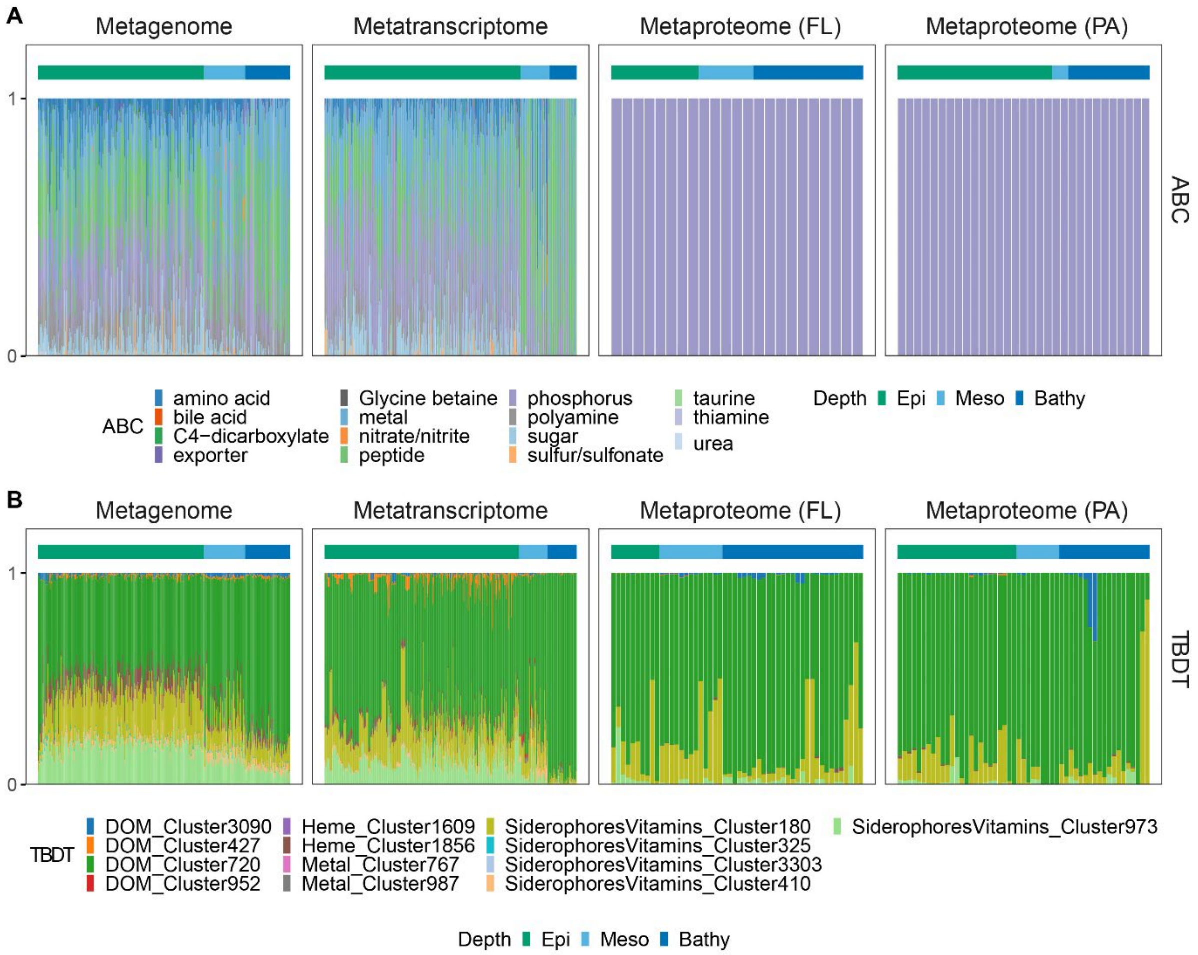

fig. S5 Substrate specificity of ABC transporters and TBDTs in Bacteroidetes reflected by metagenomic, metatranscriptomic and metaproteomic analysis A, Substrate specificity of ABC transporters. **B**, Substrate specificity of TBDTs. The *x*-axis represents samples from different stations, and the depth of the sample is indicated using the color bar on the top.

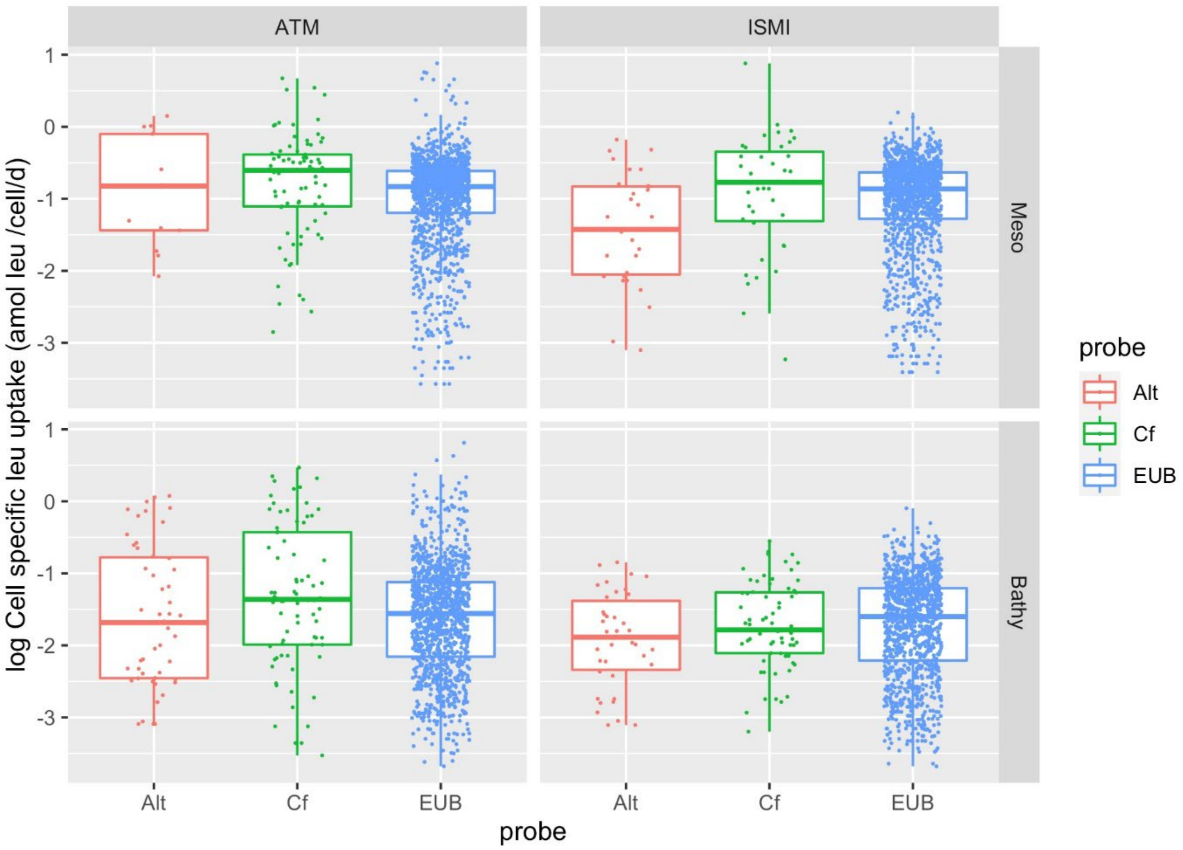

fig. S6 Cell-specific leucine uptake rate of selected taxa under *in-situ* (ISMI) and atmospheric (ATM) conditions. Alt: *Alteromonas*/*Colwellia*, Cf: Bacteroidetes, EUB: Bacteria. Original data from Amano et al. (ref 22).

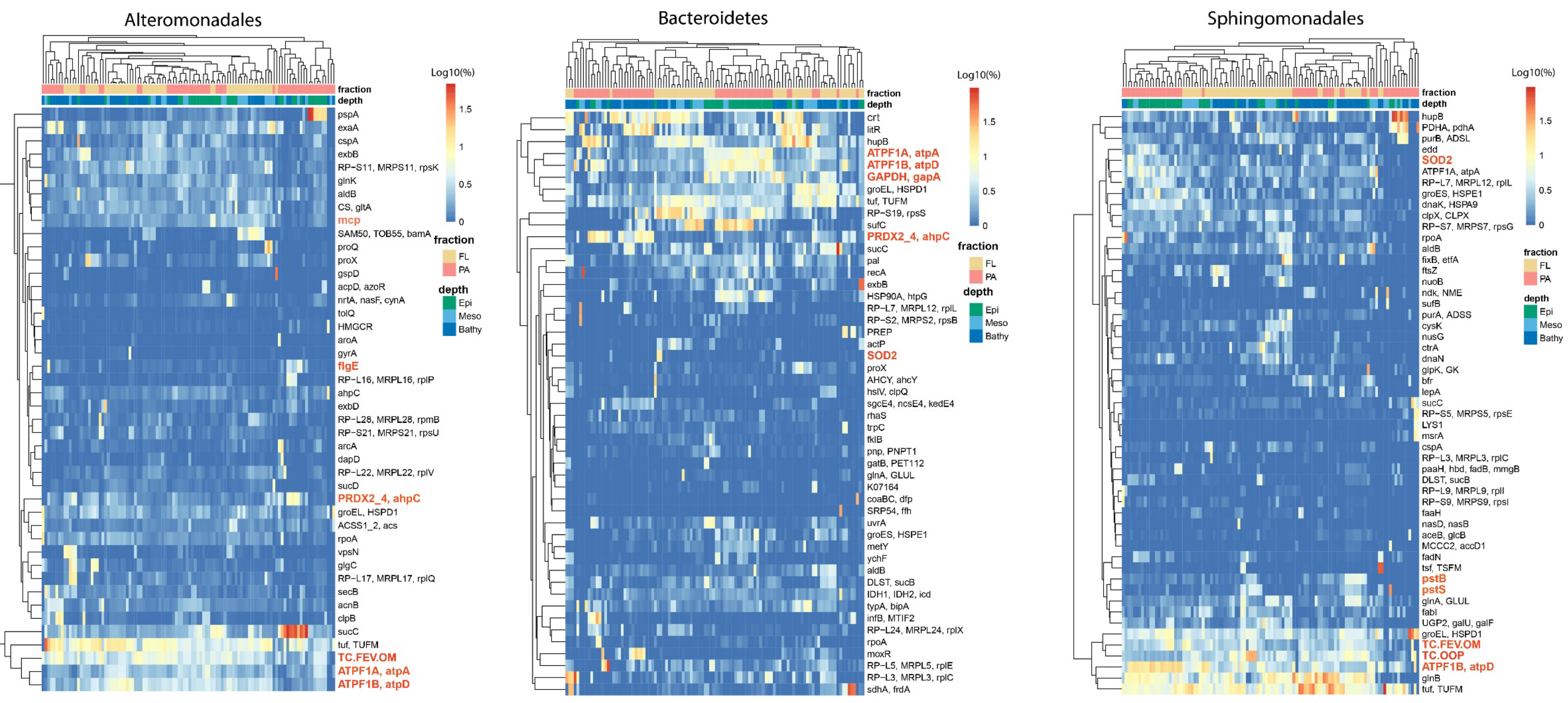
Alteromonadales
Bacteroidetes
Sphingomonadales
fraction
depth
Log10(%)
1.5
1
0.5
0
FL
PA
Epi
Meso
Bathy
pspA
exaA
cspA
exbB
RP−S11, MRPS11, rpsK
glnK
aldB
CS, gltA
mcp
SAM50, TOB55, bamA
proQ
proX
gspD
acpD, azoR
nrtA, nasF, cynA
tolQ
HMGCR
aroA
gyrA
flgE
RP−L16, MRPL16, rplP
ahpC
exbD
RP−L28, MRPL28, rpmB
RP−S21, MRPS21, rpsU
arcA
dapD
RP−L22, MRPL22, rplV
sucD
PRDX2_4, ahpC
groEL, HSPD1
ACSS1_2, acs
rpoA
vpsN
glgC
RP−L17, MRPL17, rplQ
secB
acnB
clpB
sucC
tuf, TUFM
TC.FEV.OM
ATPF1A, atpA
ATPF1B, atpD
crt
litR
hupB
ATPF1A, atpA
ATPF1B, atpD
GAPDH, gapA
groEL, HSPD1
tuf, TUFM
RP−S19, rpsS
sufC
PRDX2_4, ahpC
sucC
pal
recA
exbB
HSP90A, htpG
RP−L7, MRPL12, rplL
RP−S2, MRPS2, rpsB
PREP
actP
SOD2
proX
AHCY, ahcY
hslV, clpQ
sgcE4, ncsE4, kedE4
rhaS
trpC
fklB
pnp, PNPT1
gatB, PET112
glnA, GLUL
K07164
coaBC, dfp
SRP54, ffh
uvrA
groES, HSPE1
metY
ychF
aldB
DLST, sucB
IDH1, IDH2, icd
typA, bipA
infB, MTIF2
RP−L24, MRPL24, rplX
rpoA
moxR
RP−L5, MRPL5, rplE
RP−L3, MRPL3, rplC
sdhA, frdA
hupB
PDHA, pdhA
purB, ADSL
edd
SOD2
ATPF1A, atpA
RP−L7, MRPL12, rplL
groES, HSPE1
dnaK, HSPA9
clpX, CLPX
RP−S7, MRPS7, rpsG
rpoA
aldB
fixB, etfA
ftsZ
nuoB
ndk, NME
sufB
purA, ADSS
cysK
nusG
ctrA
dnaN
glpK, GK
bfr
lepA
sucC
RP−S5, MRPS5, rpsE
LYS1
msrA
cspA
RP−L3, MRPL3, rplC
paaH, hbd, fadB, mmgB
DLST, sucB
RP−L9, MRPL9, rplI
RP−S9, MRPS9, rpsI
faaH
nasD, nasB
aceB, glcB
MCCC2, accD1
fadN
tsf, TSFM
pstB
pstS
glnA, GLUL
fabI
UGP2, galU, galF
groEL, HSPD1
TC.FEV.OM
TC.OOP
ATPF1B, atpD
glnB
tuf, TUFM

fig. S7 Proteomic expression of Alteromonadales, Bacteroidetes and Sphingomonadales in the free-living (FL) and particle-associated (PA) fraction along the water column.

table S1 Statistics of identified transporter proteins

	ABC transporters	TBDTs
No. of predicted genes	479,317	272,981
No. of proteins	4,531	7,225
No. of peptides	7,136	17,835
No. of unique peptides	5,633	11,444
Peptide spectrum matches	57,565	93,263

table S2 Substrate classification of TBDTs

**Substrate category**	**Cluster**	**Substrates**
DOM	Cluster 3090	Chito-oligosaccharides, phytate, maltodextrin, maltose, chitin, xylan, xylose, pectin
	Cluster 720	Digested proteins, starch /malto-oligo-saccharides, chondroitin sulfate/ hyaluronic acid
	Cluster 427	Arabinose
	Cluster 952	Sucrose
Siderophore/ Vitamin	Cluster 3303	Ferric-citrate
	Cluster 410	Aerobactin, alcaligin, anguibactin, catecholates, chrysobactin, coprogen, ferrioxamine B, rhodoturolic acid, desferrioxamine, ferric malleobactin, ferric ornibactin, ferrichrome, hexylsulfate, pseudobactin A, pseudobactin M114, pyochelin, pyoverdine, rhizobactin 1021, thiamin, vibriobactin, yersiniabactin
	Cluster 973	Vitamin B12, catecholates, enterobactin, 2,3-dihydroxybenzoylserine (DHBS)
	Cluster 325	Vitamin B12
	Cluster 180	Fibronectin, thiamin
	Cluster 2835	Thiamin
Heme	Cluster 1609	Heme
	Cluster 1856	Heme
Metal	Cluster 767	Copper, Copper chelate
	Cluster 987	Nickel, Cobalt

Captions for supplementary datasets:

Supplementary dataset1: Sampling information of metagenome, metatranscriptome and metaproteome

Supplementary dataset2: Taxonomy of genes, transcripts and proteins of transporters at phylum level

Supplementary dataset3: Substrate of genes, transcripts and proteins of transporters at phylum level

Supplementary dataset4: Taxonomy of genes, transcripts and proteins of transporters at order level for Alpha- and Gammaproteobacteria

Supplementary dataset5: Sequences used for TBDT substrate identification
